# Understanding Racial Disparities in Prostate Cancer: A Multifaceted Approach

**DOI:** 10.1002/cam4.70979

**Published:** 2025-05-30

**Authors:** Charles Cobbs, Gregory T. Chesnut, Ayesha A. Shafi

**Affiliations:** ^1^ Center for Prostate Disease Research, Murtha Cancer Center Research Program, Department of Surgery Uniformed Services University of the Health Sciences Bethesda Maryland USA; ^2^ Urology Service, Department of Surgery Walter Reed National Military Medical Center Bethesda Maryland USA; ^3^ Henry M. Jackson Foundation for the Advancement of Military Medicine Inc. Bethesda Maryland USA

**Keywords:** access to healthcare, genomic studies, prostate cancer, racial disparities, socioeconomic status

## Abstract

Prostate cancer (PCa) remains a significant public health challenge in the United States, disproportionately affecting African American (AA) men, who face higher incidence rates, more aggressive disease, and elevated mortality compared to Caucasian American (CA) men. This review explores the multifactorial underpinnings of these disparities, integrating genomic, socioeconomic, environmental, and systemic contributors. Genomic analyses reveal that AA men harbor distinct molecular alterations, including higher frequencies of FOXA1, BRAF, and CHD1 mutations, as well as DNA damage repair defects, highlighting the critical need for population‐specific precision medicine. Immune‐oncologic pathways and stromal interactions within the tumor microenvironment further underscore biological differences driving aggressive disease phenotypes. Concurrently, adverse social determinants—including limited access to care, lower PSA screening rates, delayed treatment, medical mistrust, and underrepresentation in clinical trials—contribute to poorer outcomes. Despite these challenges, evidence from equal‐access healthcare systems indicates that when provided equitable treatment, AA men can achieve outcomes comparable to or better than their CA counterparts. This review emphasizes actionable strategies to reduce disparities, including increasing AA representation in clinical trials, enhancing culturally competent patient‐provider communication, improving access to early detection and high‐quality care, and expanding community‐based outreach initiatives. A holistic, interdisciplinary approach is essential to dismantle systemic barriers and achieve health equity in prostate cancer outcomes.

## Graphical abstract

Prostate cancer disproportionately affects African American men. Genomic studies reveal distinct mutational burdens in AA men, highlighting the need for race‐specific therapies. However, limited representation in research hinders progress. Addressing disparities requires diverse clinical trials, improved access to care, and tackling socioeconomic inequities.

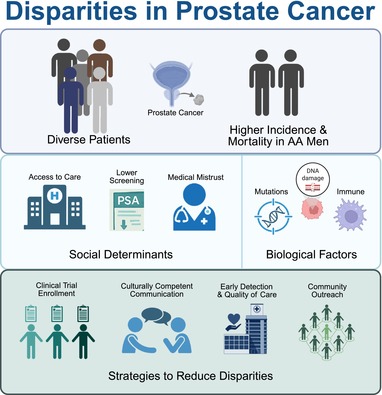

## 1 Introduction

**FIGURE 1:**
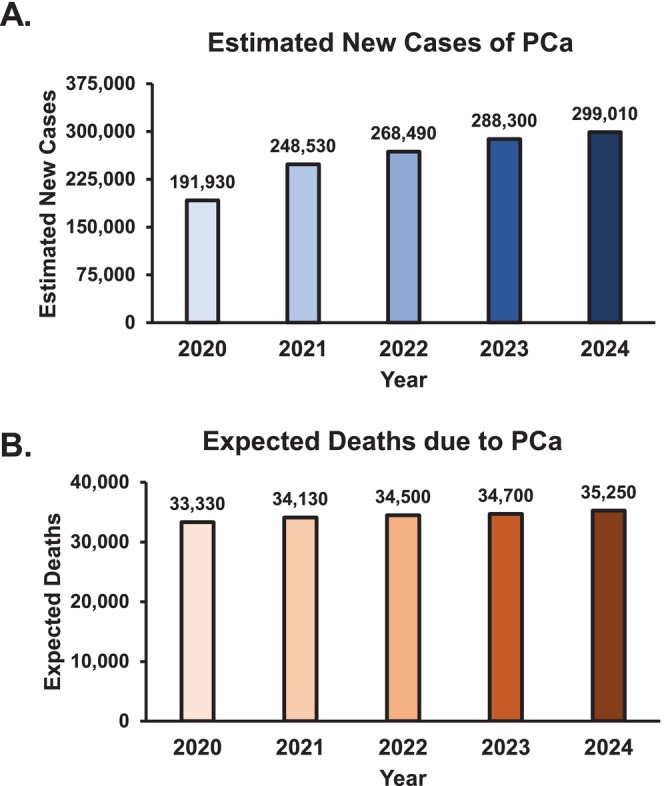
Prostate cancer (PCa) cases and deaths in AA and CA men. (A) Estimated new PCa cases and (B) expected deaths due to PCa by year in AA and CA men in the USA from 2020 through 2024 provided by American Cancer Society (ACS) statistics.

Prostate cancer (PCa) is the second leading cause of cancer‐related death among men in the United States [1, 2]. In recent years, both the incidence of nonregional disease at diagnosis and PCa‐specific mortality have risen. New PCa cases have risen from 191,130 in 2020 to 299,010 in 2024 (Figure 1A), and estimated deaths have increased from 33,330 in 2020 to 35,250 in 2024 (Figure 1B) [1, 3, 4, 5, 6]. While advancements in screening and diagnostic techniques may contribute to the rise in detected cases, the concurrent increase in PCa‐related mortality suggests a growing burden of clinically significant disease.

**FIGURE 2:**
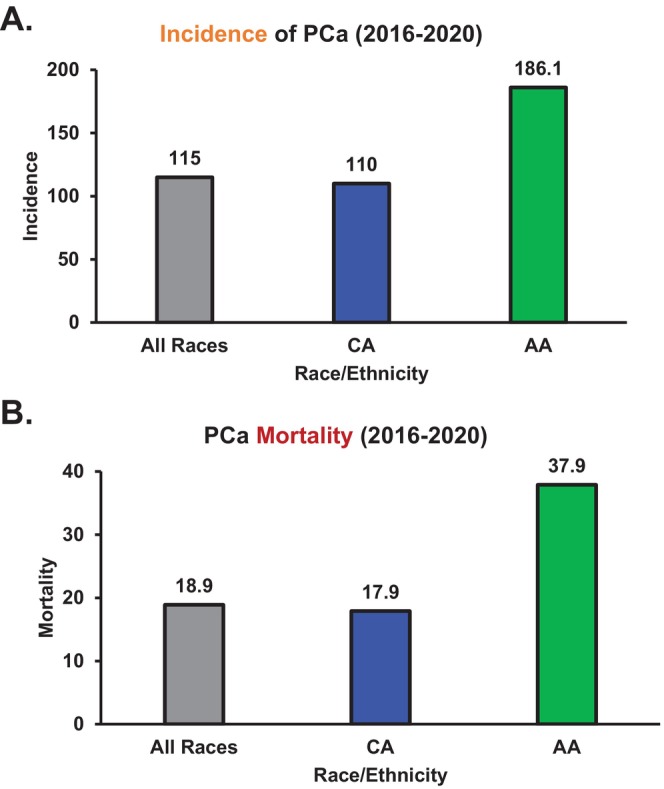
Prostate cancer (PCa) statistics in AA and CA men. (A) Incidence of PCa and (B) mortality rates in AA and CA men in the USA from 2016 through 2020 provided by American Cancer Society (ACS) statistics.

This burden is particularly pronounced among African American (AA) men, who experience a 1.5‐fold higher incidence and 2.5‐fold higher mortality rate compared to Caucasian American (CA) men (Figure 2) [7, 8]. AA men have the highest PCa incidence in the United States, with 186.1 new cases per 100,000, and they are more likely to develop aggressive disease at earlier ages and across all disease stages [1, 2, 8, 9]. Consequently, survival rates at 1‐, 5‐, and 10‐year post‐diagnosis are significantly lower for AA men than for CA men [1, 2]. These disparities underscore the urgent need for race‐specific predictive models incorporating clinical, molecular, and biological data to improve risk stratification, treatment personalization, and patient outcomes.

Given these trends, addressing the disparities in PCa outcomes requires a multifaceted approach, including improved screening strategies, increased representation of AA men in clinical research, and enhanced understanding of molecular drivers of aggressive disease in this population. Personalized screening and targeted interventions are essential to mitigating the disproportionate impact of PCa on AA men.

Many biomarkers can suggest the presence of PCa, such as the TMPRSS2‐ERG fusion gene, non‐coding RNA (PCA3), or kallikrein, which are included in the basic prostate health index or 4K tests [10]. However, the prostate‐specific antigen (PSA) test is the strongest clinical indicator to date [11]. PSA is the standard test but is relatively non‐specific and imprecise, given that both benign and malignant processes can cause elevated levels in blood [11]. However, despite these concerns, it remains the single most useful tool for early PCa screening, providing individuals with the best chance of survival [11, 12, 13].

Screening and clinical research to develop early detection methods and discover novel biomarkers of aggressive disease are the best ways to identify PCa amenable to treatment while limiting overtreatment of indolent disease. As stated earlier, PSA remains the gold standard for early detection of PCa, which level 1 evidence shows results in increased cancer‐specific survival [11]. However, the prevalence of PSA screening is lowest in the AA demographic despite PCa incidence and mortality being the highest [7]. As a result, the United States Preventive Services Task Force (USPSTF) lacks specific recommendations for screening AA males, citing a lack of data on the risks and benefits of screening among this population. Furthermore, AA men are also underrepresented in key PCa trials, underscoring the lack of racial disparity in discerning mechanisms of action and enhancing therapeutic impact to combat this lethal disease [7, 14, 15]. Several socio‐economic factors contribute to the underrepresentation of AA men in clinical settings, including historic distrust in the medical system, lack of access to trials, and lack of awareness of trials to participate in [7]. Thus, these factors contribute to the racial disparity and gap in treatment for AA men.

Genomic, environmental, socio‐economic, and institutional factors all contribute to disparity in diagnosis, treatment, and outcomes of PCa. While these factors are multifaceted and complex, institutionalized racism continues to be a central driver. Although significant research has been conducted on PCa, particularly in AA men, a critical gap remains in translating these findings into actionable strategies to address racial disparities in PCa outcomes. This review bridges that gap by providing a comprehensive overview of the factors contributing to these disparities, including genetic, environmental, and social determinants. This review serves as an interdisciplinary perspective by integrating insights from diverse fields, including genetics, epigenetics, sociology, and public health, to provide a holistic understanding of PCa disparities. Furthermore, in this review, we offer practical recommendations for clinicians, researchers, and policymakers to address disparities and improve outcomes for AA men with PCa. By highlighting resources and strategies for outreach and community engagement, this review aims to empower AA men and their families to take control of their health. Thus, this review serves as a valuable resource for researchers, clinicians, and policymakers to advance the field of PCa research and improve the health of AA men.

## 2 Genomic Predispositions for PCa in AA Males

In research surrounding PCa, genetic ancestry is a common factor of interest. This is due to the high rate of heritability and the observation of men of African ancestry living in the United States carrying a high incidence of disease [7, 9, 15, 16]. Humans broadly share approximately 99% of the genome; however, there are informative markers of ancestry that can distinguish ancestry [7]. Studies that focus on this aspect of PCa typically utilize genomic analysis to determine genetic race, allowing them to shift from the self‐report mechanism [15]. Historically, PCa studies lacked adequate genetic ancestry, and researchers were forced to rely on self‐reported race or investigator‐assigned race, which is not always an accurate description. Race is widely used in biomedical research as a proxy for many risk factors; however, the lines are typically blurred when trying to decipher what is meant by race in the context of each study. Race alone is not a cause of pathology. If using race as a proxy for an unmeasured variable, describing and discussing the potential limitations is necessary. Genetic ancestry, racism, discrimination, and socioeconomic status are all common factors for which race is a proxy [7]. It is important to recognize when analyzing data from studies of this category that in the United States, race is a social classification based strictly on phenotype and cultural factors. It is not a biological variable [7]. Therefore, it is important to define this in all biomedical research surrounding race and distinguish the proxy it is being utilized for and its potential limitations [17].

Tumorigenesis and genetic profiling in PCa demonstrate racial variability, with distinct mutational burdens that could serve as novel biomarkers and therapeutic targets (Table 1). These differences highlight the potential for race‐specific precision medicine. One key finding is the higher prevalence of FOXA1 mutations in AA men compared to CA men (18.6% vs. 11.9%) [15, 18]. FOXA1, a pioneer factor in androgen receptor (AR) signaling, plays a crucial role in prostate cell growth and survival [18]. Given its importance in PCa progression, preclinical modeling of FOXA1 alterations in AA men is essential for developing targeted therapies.

**TABLE 1:** PCa resources for AA men.

Resource	Summary	Link
ZERO	ZERO is a nonprofit organization whose mission is to end PCa and help those impacted. This is done via research advancement, community support, and creating solutions to achieve health equity	https://tinyurl.com/2zr2m9en
Prostate Cancer Foundation (PCF)	The PCF funds promising research on biology and treatment of PCa while also providing resources for those currently affected by PCa	https://tinyurl.com/bddrn68e
Schaufeld Program for Prostate Cancer in Black Men	The Schaufeld Program for Prostate Cancer in Black Men is a scientifically founded, community‐based program that aims to reduce impact of PCa among Black men	hhttps://tinyurl.com/3y2dtnd9
PHEN	PHEN's mission is to eliminate the African American prostate cancer disparity, and to increase the overall support and resources for a war on prostate cancer that will lead to a cure for the disease for the benefit of all men	https://tinyurl.com/bdcmpaap
CAAPP	CAAPP aims to encourage AA males aged 40+ to take PSA blood tests, help men better understand test results and their screening experience, ad to increase access and build relationships with primary care physicians	https://tinyurl.com/mr3jn3kp
Prostate Health Awareness Project	This is an educational project that comprises of a booklet and video that presents balanced information and the risks and benefits of PCa screening. The goal is to enhance knowledge in the decision‐making process for PCa screening among AA men	https://tinyurl.com/dhvhh67h

Another significant molecular alteration is the BRAF mutation, a key activator of the mitogen‐activated protein kinase (MAPK) pathway, which drives tumorigenesis [19, 20, 21]. Studies of metastatic PCa have shown higher rates of AR and actionable genetic mutations, such as BRAF and DNA repair gene alterations, in AA men compared to CA men [15, 19, 20, 21, 22, 23]. These findings reinforce the need to prioritize AA representation in clinical trials to refine prognostic models and therapeutic strategies.

Beyond individual gene mutations, broader chromosomal susceptibility factors also contribute to racial disparities in PCa. Chromosome 8q24, a major PCa susceptibility locus, interacts with the MYC proto‐oncogene and is significantly altered in AA men [9, 16, 24]. Targeting this region could yield promising therapeutic advances. Similarly, CHD1, a chromatin‐remodeling protein involved in DNA repair and gene transcription, is deleted nearly three times more frequently in prostate tumors of AA men (29.7%) compared to those of European Ancestry (EA) men (11%) [25]. CHD1 loss is associated with DNA repair defects, AR dysfunction, and aggressive disease progression, making it a potential biomarker for therapeutic response in AA patients [26, 27, 28].

AR remains a central driver of PCa, and missense mutations in AR occur at higher frequencies in AA men compared to CA men [15, 29]. A notable example is the T877A gain‐of‐function mutation, which has been linked to distinct gene expression profiles and differential survival outcomes [29, 30]. This mutation also interacts with DNA damage repair (DDR) proteins such as BRCA1/2, suggesting a potential therapeutic role for DDR‐targeting agents like PARP inhibitors in AA men [29].

The genetic landscape of PCa reveals significant racial variability, with key mutations and chromosomal alterations contributing to disparities in disease progression and treatment response. The higher prevalence of FOXA1 mutations, BRAF alterations, and DNA repair gene defects in AA men underscores the need for tailored therapeutic strategies and improved clinical trial representation. Additionally, broad chromosomal susceptibility loci such as 8q24 and deletions in CHD1 further highlight the complex genetic architecture influencing PCa disparities. The AR remains a central player, with recurrent missense mutations, including T877A, potentially altering treatment response. While these genetic modifiers provide critical insights, numerous additional alterations have been identified in AA men, but their comprehensive discussion is beyond the scope of this review. Continued research and increased inclusion of AA patients in genomic studies are essential for refining precision medicine approaches and ultimately improving PCa outcomes in this population. However, these efforts remain hindered by the underrepresentation of AA men in genomic studies. Expanding participation through targeted outreach and policy initiatives is critical to refining precision medicine approaches and improving PCa outcomes in AA communities.

Together, these findings illustrate a complex genetic landscape that contributes to disparities in PCa progression and treatment response. The higher prevalence of FOXA1 mutations, BRAF alterations, CHD1 deletions, and DDR gene defects in AA men highlights the need for tailored therapeutic strategies and broader clinical trial inclusion. While this review cannot cover all known mutations, it is clear that many additional genomic differences exist. Addressing the persistent underrepresentation of AA men in genomic studies—through targeted outreach, community engagement, and policy reform—is essential for realizing the full potential of precision oncology and achieving equity in PCa outcomes.

## 3 Polygenic Hazard Scores and Risk Variants

Polygenic hazard scores are genetic risk models that aggregate the effects of multiple genetic variants to predict the risk of developing PCa [31]. For AA men, specific improvements have been made to these scores to enhance their predictive accuracy. For instance, the PHS46 + African model, which includes 46 SNPs identified in European populations and three additional SNPs (rs76229939, rs74421890, and rs5013678) located on chromosome 8q24, has shown substantial improvements in predicting the age at diagnosis of PCa in AA men [31].

Risk variants specific to AA men have also been identified. A study identified nine novel susceptibility loci for PCa, with seven being either unique to or more common in men of African ancestry [32]. These include an African‐specific stop‐gain variant in the prostate‐specific gene anoctamin 7 (ANO7). Additionally, a multi‐ancestry polygenic risk score (PRS) incorporating 278 risk variants has been shown to be effective in stratifying PCa risk and differentiating between aggressive and non‐aggressive disease in men of African ancestry.

These findings underscore the importance of including diverse populations in genetic studies to improve the accuracy and clinical utility of genetic risk scores for PCa, particularly in high‐risk groups such as AA men.

## 4 Tumor Microenvironment and Immune‐Oncologic Pathways in African American Men

Distinct immune‐oncologic pathways and stromal interactions in the tumor microenvironment (TME) of AA men with PCa may contribute to the aggressive nature of the disease in this population. Several studies have identified key immune signatures and stromal factors that differentiate AA prostate tumors from those of European American (EA) men.

One study in particular found that AA prostate tumors exhibit significant enrichment of major immune‐oncologic pathways, including proinflammatory cytokines, IFNα, IFNγ, TNFα signaling, interleukins, and epithelial‐mesenchymal transition (EMT) [33]. These pathways are notable for their roles in promoting tumor growth, progression, and immune evasion. Proinflammatory cytokines enhance inflammation, which supports tumor development by increasing angiogenesis (blood supply formation), promoting DNA damage, and facilitating immune escape. IFNα and IFNγ are essential for antiviral and antitumor immunity: IFNγ activates macrophages and boosts antigen presentation, while IFNα enhances immune detection of tumor cells. However, sustained interferon signaling can paradoxically lead to immune exhaustion and allow tumors to escape immune control. TNFα, while capable of inducing apoptosis in some contexts, often promotes tumor growth in prostate cancer by sustaining chronic inflammation and fostering a tumor‐friendly microenvironment. Interleukins (e.g., IL‐6, IL‐8, IL‐18) regulate immune cell proliferation and migration, and several are linked to more aggressive PCa phenotypes and worse prognoses. EMT, a process by which epithelial cells acquire migratory and invasive properties, is a hallmark of metastasis and is frequently upregulated in aggressive prostate tumors. The significant enrichment of these major immune‐oncologic pathways in AA tumors may help explain the more invasive disease course observed in this population [33].

Stromal interactions also play a crucial role in PCa progression among AA men. A further study demonstrated that stromal cells in the AA TME promote tumor progression by increasing levels of pro‐inflammatory molecules such as BDNF, CHI3L1, DPPIV, FGF7, IL18BP, IL6, and VEGF [34]. These stromal‐derived mediators enhance tumor proliferation and motility, reinforcing the need for targeted research into the stromal contributions to PCa disparities.

Further emphasizing the importance of the tumor‐adjacent stroma, one study reported that the majority of differentially expressed genes between AA and CA PCa tissues were found in the stroma rather than the tumor itself [35]. They identified downregulation of extracellular matrix, integrin family, and EMT pathways in the stroma of AA men, which may impact tumor progression and metastatic potential.

Collectively, these findings highlight distinct immune and stromal interactions in AA‐derived PCa, underscoring the need for targeted research and therapeutic strategies that address the unique biology of this vulnerable population. Expanding studies on the TME, immune responses, and stromal contributions in AA PCa is critical to developing race‐specific precision medicine approaches and improving outcomes in high‐risk groups.

## 5 Epigenomics and the Supporting Factors that Impact Biology

Historical and ongoing inequities paired with structural racism have been reflected in the statistic that AA men, on average, have lower income, education, housing, and employment rates in comparison to CA males [36, 37]. Epigenetics is founded on the idea that genes and environment are known to interact and that some patients may be genetically predisposed to respond to environmental contributions more than others. Low socioeconomic status (SES) is a well‐known risk factor for poor healthcare access, hazardous habits, and many diseases, including PCa [37]. Low SES has even been dubbed a driver for worsening PCa prognoses [37, 38]. As a result, it can be suggested that unique biological changes and disparate treatment outcomes in AA males with PCa are rooted in adverse environments.

Studies have shown a strongly positive association between worsening environmental quality and the odds of having a metastatic PCa at diagnosis [39]. A 2020 study utilized the environmental quality index (EQI) to determine the association between environmental quality and PCa staging at diagnosis [39]. This measurement tool summarizes environmental data into an ecological index at the United States County level and stratifies data by five different exposure domains: air, water, land, built, and sociodemographic [39]. The study's results showed that not only were AA males living in poorer‐quality environments, but they were also presented with more aggressive forms of PCa [39]. These findings parallel other studies that have suggested that men who live in neighborhoods with an unfavorable SES are more likely to be diagnosed with high‐grade PCa [39, 40]. Statistically, it is AA males who are enduring this residential circumstance at a higher rate than CA [39, 40]. This leaves the AA demographic at a higher predisposition for tumorigenesis and disease severity. These studies highlight a relationship between residential quality by way of SES and cancer vulnerability and disease severity.

PCa is one of the most common human malignancies that arises through genetic and epigenetic alterations [41]. There are a plethora of genes that can undergo aberrant hypermethylation in PCa, and these methylation profiles tend to differ among AA and CA men [41]. A complex combination of environmental, socioeconomic, and genetic variations may all contribute to the disparities of incidence and mortality in PCa. Differences in the distribution of aberrant methylation may contribute to differential cancer health disparities among varying demographics. An early study investigated differences in DNA hypermethylation of three different *genes* in tumor tissues from AA and CA men using real‐time methylation–sensitive PCR [42]. The study identified that the frequency of methylation of one of the three genes, in this case CD44, was higher in AA men compared with CA men [42]. A better understanding of the epigenetic changes in PCa is likely to contribute to improved diagnosis, clinical management, and better outcomes.

PCa is one of the most common human malignancies that arises through genetic and epigenetic alterations [43]. Methylation, an epigenetic modification, plays a crucial role in the development of PCa by silencing tumor suppressor genes and allowing for unchecked cellular proliferation. A growing body of research highlights that methylation profiles in PCa differ between AA and CA men, potentially contributing to disparities in incidence and mortality [43]. Specifically, aberrant hypermethylation of genes such as CD44 has been shown to occur more frequently in tumor tissues from AA men compared to their CA counterparts [44]. This increased frequency of methylation in AA men may lead to a higher likelihood of mutations and genetic instability, which could contribute to the development of PCa at a higher rate in this population. These differences in methylation patterns are likely influenced by a complex interplay of environmental, socioeconomic, and genetic factors. As such, a better understanding of the epigenetic changes in PCa, particularly the role of methylation, could provide insights into the mechanisms underlying cancer health disparities and pave the way for improved diagnosis, clinical management, and outcomes for AA men.

Socioeconomic status sits as a premiere determinant of mortality in cancer patients. Findings from the National Program of Cancer Registries Patterns of Care Study show that compared to CA, AA have a higher risk of mortality after a disease diagnosis [37]. Further results indicated an association between low SES and increased mortality that was more apparent among racial/ethnic minorities, which suggests that interacting forces between SES and race/ethnicity contribute to cancer disparities [37]. There is also compelling evidence that lower socioeconomic status in AA men and disparities in treatment are essential drivers of observed differences in survival. Survival in the United States is worse in AA men diagnosed with low‐grade PCa but not with high‐grade disease [45]. Such provides evidence of disparities in care as true, low‐grade disease should not be associated with mortality. This paints a picture of PCa in the same light as countless other topics in medicine. There are factors of disease burden where racial differences attribute to significantly different outcomes and disparate care; revealing an unfortunate implication of structures in society and the medical field where racism still exerts its noxious effects.

## 6 Active and Current Clinical Trials

Several active and successful clinical trials have specifically examined treatment outcomes for Black men with PCa, highlighting both the efficacy of current therapies and the need for greater representation in research.

A notable prospective trial evaluating abiraterone acetate plus prednisone in Black and White men with metastatic castration‐resistant prostate cancer (mCRPC) found that Black men had similar radiographic progression‐free survival (rPFS) and overall survival (OS) to White men, with a trend toward longer time to PSA progression and greater PSA declines [46]. However, Black men experienced higher rates of side effects, including hypertension, hypokalemia, and hyperglycemia, emphasizing the importance of understanding race‐specific treatment responses and toxicity profiles.

Another study examining disparities in systemic therapy outcomes for advanced PCa found that Black men had similar or even better outcomes than White men when treated in equal‐access settings [47]. This suggests that nonbiological factors, such as healthcare access and delivery, play a significant role in racial disparities, reinforcing the need for equitable treatment opportunities.

The Decipher genomic classifier study (NCT02723734) is a prospective trial that includes AA men to evaluate genomic risk reclassification and identify those with high genomic risk of early metastatic disease [48]. By integrating genomic classifiers with clinical risk assessments, this study aims to refine treatment strategies for AA men with localized PCa, ensuring that personalized therapeutic approaches account for distinct genetic and molecular features.

Additionally, a phase II trial focused exclusively on Black men with mCRPC treated with abiraterone acetate plus prednisone confirmed a comparable safety and efficacy profile to predominantly White populations [49]. However, low accrual rates in this trial highlight the persistent challenge of underrepresentation in PCa research.

These findings underscore the critical need to increase Black male participation in clinical trials to ensure that treatment efficacy, safety, and toxicity are fully understood across diverse populations. Expanding outreach, improving trial accessibility, and addressing systemic barriers will be essential to advancing precision medicine and reducing racial disparities in PCa outcomes.

## 7 Access to Medical Care and Patient–Doctor Relationships

Reported by numerous studies, access to health care and its social, economic, and behavioral correlates are strongly associated with racial disparities in PCa [14, 43, 44]. A 2021 Massachusetts study reported that racial differences in mortality persisted even after adjusting for sociodemographic and known PCa prognostic factors [50]. These mortality differences were because AA men were 40% less likely to receive definitive treatment (defined as prostatectomy or radiation therapy within 90 days of diagnosis) when compared to CA men [50]. This same study later assessed receipt of definitive treatment and its relationship to insurance status, and results suggest that when AA men are insured, they derive a larger increase in treatment rates than CA men [50]. Other studies have shown that the treatment and care received by AA males differed from CA in terms of quality [38, 43, 44, 51]. Further, many publications have revealed that AA men have worse access to PCa screening, appropriate follow‐up, and receipt of definitive therapy [51]. Thus, the inequity in clinical care across racial cohorts is concerning and needs to be minimized to enhance patient outcomes.

Despite socioeconomic disadvantage, many studies suggest that AA men may fare as well or even better than CA men when equivalently treated. In the military's equal access healthcare system, for example, AA men show equivalent outcomes to CA patients, and both groups outperform those men treated in the general population. Specifically, a study assessing 5‐year survival of men with advanced PCa tumors revealed that patients in the military health system had improved survival compared to their counterparts in the United States general population, and the survival advantage was consistently observed in all patients, regardless of race [52]. A retrospective cohort study revealed that in the equal access health setting, specifically the military health system, despite AA men having shorter survival time from radical prostatectomy to biochemical recurrence (BCR), the group showed comparable survival time from BCR to metastasis and metastasis to overall death [53]. These military‐based studies support the concept that AA men with PCa can do as well or better than CA men with PCa if they receive the same high‐quality care. Further, active surveillance (AS) is the standard of care for most men with favorable‐risk, low Gleason grade PCa. Several studies have shown that race does not significantly impact the oncologic outcomes of men on AS for low‐risk PCa. For instance, a study by Pincus et al. demonstrated that race was not predictive of Gleason grade progression, AS discontinuation, or biochemical recurrence in a cohort with a majority of AA men [54]. Similarly, Deka et al. found that AA men had a higher incidence of disease progression and definitive treatment compared to CA men, but no significant differences in metastasis or PCa‐specific mortality [55]. These studies are highlighted in Table S1. This illustrates the need to provide a historically vulnerable demographic with adequate treatment and access to said treatment, as it is evident that elevated treatment quality for AA men directly impacts survival.

Utilization of PSA screening plays an important role in PCa disparities. As mentioned previously, AA men are less likely to undergo PSA screening in comparison to CA men; as a result, diagnosis is frequently delayed, causing poor outcomes [7, 8, 56]. However, in scenarios where PSA screening is equally accessible regardless of race, rates of screening may differ less, thus limiting this disparate factor [57, 58]. A cohort study conducted by the Center for Prostate Disease Research (CPDR), a multi‐center national database associated with Walter Reed Army Medical Center and the Uniformed Services University of Health Sciences, evaluated PCa survival in active surveillance patients [43, 58]. Active surveillance refers to a type of management that involves the close monitoring of a patient's condition without treatment until testing shows it is indicated. Growing concerns regarding the overtreatment of PCa have led to an increased interest in this form of monitoring and treatment [43]. All patients included in the study were eligible for military health care regardless of education, income, or place of residence [43]. This provided a relative uniformity concerning SES in the cohort. Among 886 eligible patients, 21% were AA males [43]. Despite racial differences in risk characteristics and secondary treatment patterns, study results showed no racial disparity in overall survival [43]. In sum, removing economic barriers and providing access to medical care promotes clinical equity and enhances early detection, decreasing disease burden and improving therapeutic regimens to combat PCa.

A significant factor contributing to disparities in PCa treatment is the persistent communication gap between patients and providers, largely influenced by physician mistrust among AA men. Studies have shown that AA men exhibit significantly lower levels of trust in the healthcare system compared to CA men, leading to delayed care, misperceptions about disease severity, and reduced engagement in shared decision‐making [7, 59, 60, 61].

The Group‐Based Medical Mistrust Scale (GBMMS), a validated 12‐item scale measuring suspicion of healthcare providers, expectations of racial discrimination, and perceived support from physicians, illustrates this disparity [59, 62, 63]. In one study, AA men had a significantly higher mistrust score (mean: 26.3, SD: 7.5) compared to CA men (mean: 21.1, SD: 5.9), highlighting a pronounced difference in attitudes toward the healthcare system [59].

This mistrust directly impacts treatment decisions and patient–provider communication. A study assessing attitudes toward PCa care and shared decision‐making before PSA testing found that even when provided with treatment information, AA men remained more confused than CA men [64]. Additionally, AA men with aggressive, high‐risk PCa were more likely to misperceive the severity of their disease, resulting in a lower likelihood of pursuing treatment [65]. This suggests that ineffective communication and inadequate patient education further contribute to treatment disparities.

However, research indicates that enhancing patient engagement in treatment decision‐making can significantly improve patient–physician trust. The CPDR Treatment Decision‐Making Study found that when patients actively participated in discussions about their treatment options, they were more likely to view healthcare providers as a reliable source of information [66]. Removing barriers to mistrust and ensuring patients have access to clear, culturally competent medical communication can help bridge this gap and improve PCa outcomes for AA men.

Addressing this issue requires targeted interventions to rebuild trust, including provider training in culturally sensitive communication, increased representation of AA men in clinical research, and community‐driven efforts to improve health literacy. Strengthening patient‐provider relationships is essential to ensuring that AA men receive equitable, effective, and patient‐centered PCa care.

The road to medical breakthroughs involves clinical trials. Currently, AA men are grossly underrepresented in said trials [7]. Efforts focused on increasing AA representation in clinical trials are vital. Further studies are needed to identify barriers to trial participation among AA patients and to evaluate outreach and educational approaches which account for social, socioeconomic, systemic, and historical factors that lead to underrepresentation.

Strides have been made with the Prostate Cancer Ambassador program and further efforts are being made by states with more rural populations, such as South Carolina, where AA men have typically been difficult to reach to create a decision guide of culturally appropriate materials that is targeted toward the AA men [67, 68]. The Ambassador Program was designed to address disparities by utilizing community health advisors who closely resembled the populations they served, both in racial and cultural background. This representation likely played a role in fostering trust and engagement within AA communities, making it a promising model for improving PCa awareness and participation in genomic studies. However, the effectiveness of such programs may also depend on the racial and cultural background of healthcare providers themselves. Studies have suggested that racial concordance between patients and providers can enhance trust, improve communication, and lead to better decision‐making [69]. Future efforts should consider not only community‐driven ambassador programs but also the role of provider‐patient racial concordance in mitigating mistrust and encouraging equitable healthcare engagement.

To date, there have been many efforts to recruit AA men into studies and repair the patient‐provider relationship. A study analyzed the methods of a clinical trial studying different PCa treatments utilized to increase AA recruitment. This clinical trial was so successful in recruitment that 41% of the patients examined identified as AA [70]. The lead researchers of the trial proposed that improving recruitment of AA and other non‐white groups to clinical trials stems from targeting the needs of the population of interest [70]. It is well known that historically AA men represent a lower SES than CA men; as a result, providing financial assistance and positioning clinical trials in areas closer to the population base can remove a key barrier to trials [70]. Moreover, this successful clinical trial had a diverse group of healthcare professionals and researchers as leaders of the study. This improved cultural and social alignment and resulted in an increased trust and rapport between patients and the conductors of the trial [70]. Lastly, the study emphasized community involvement in disseminating information about the clinical trial. Clinical trial leaders provided educational materials in formats that were palatable to all education levels and were adjusted to fit racially diverse populations [70]. Collectively, this study showcases the importance of these factors working together to enhance racial disparity in clinical trials, improve community trust in the healthcare system, and remove socioeconomic barriers to improve patient care.

Beyond socio‐economic factors and mistrust in the medical system, the type of healthcare facilities available to patients may also influence PCa treatment. Several studies have shown that despite having similar clinical characteristics, AA men were unlikely to receive the necessary PCa treatment and more likely to experience delays in diagnosis compared to their CA counterparts [7, 8, 71]. That said, in a study analyzing the National Cancer Institute‐designated Comprehensive Cancer Center, when adjusting for the use of equal‐access healthcare systems (i.e., Veteran's Affairs Health System), Medicare insurance, or receipt of care in clinical trials, disparities in the likelihood or time to treatment were no longer reported [7, 8, 72]. Some studies using equal‐access trial designs report that AA men were more likely to receive treatment and to have better outcomes compared with CA men [8, 73]. Comprehensively, these studies further indicate that providing AA men with quality health facilities can impact a disease burden that has consistently implicated the AA demographic.

While improved outreach, trial enrollment, and treatments are important once prostate cancer is diagnosed, improved and personalized screening programs remain the first step in improving PCa outcomes. Without understanding the increased risk among AA men to develop clinically significant PCa, it is impossible to expect that this population can make an informed decision to get screened [14, 74]. Physicians must provide comprehensible information about the advantages and disadvantages of the screening and treatment options for PCa for patients to align the medical care options with their values. An effort to execute this has been seen with the Prostate Cancer Ambassador Program [68]. This health advisor model has been used to educate individuals on PCa risks and symptoms, advise for informed decision‐making for PSA screening, and how to deliver information to their communities [68]. The pilot program trained 32 ambassadors in predominantly AA communities, and in total, these ambassadors reached 355 individuals in their communities with information on PCa [68]. The success of this program suggests that AA individuals are willing and open to learning about disease courses and treatment options, but it should be done in a digestible manner conducted by individuals who are integrated into the community.

## 8 Conclusion

AA men are disproportionately affected by PCa with earlier presentation, more aggressive disease, and higher mortality rates versus other racial groups [1, 2, 7]. Genetic, socioeconomic, and systemic factors likely contribute to this disparity. A major limitation to understanding genetic contributions to diagnosis, prognosis, and treatment is a lack of population‐specific sample size of AA men at risk for or diagnosed with PCa [15, 29, 75]. AA men are not enrolling in clinical trials at the necessary rate to make informed scientific discoveries [7]. As a result, AA men are being diagnosed later, being treated later, and in turn, dying at higher rates than CA men [2, 7].

Factors limiting AA participation in screening and treatment trials include mistrust of the health system, stigma regarding seeing a physician, and poor physician‐patient communication. Further, economic disparities that the community faces provide an immediate barrier to adequate treatment and influence the decision of whether or not even to seek out medical intervention [14, 37]. Such obstacles perpetuate the health disparity and contribute to preventable death. Much data and reviews have been published regarding PCa treatment, or lack thereof, for AA males; however, very few, if any, manuscripts have bridged the results of research with resources that directly mitigate the health equity complications discussed. This review focused on different factors, including genetic, environmental, and social contributions to the racial disparities seen in PCa, while also providing together effective resources that target treatment and prevention of PCa in AA men. We aimed for this review to serve as an encompassing source of knowledge providing both resources for outreach and community engagement, while also providing scientific insight into the racially driven genomic predispositions and epigenetic alterations as potential therapeutics. Further, this review also details the barriers that exist in access to medical care and the distrust in patient–doctor relationships, while providing tools to improve these aspects and progress forward.

Many factors contribute to PCa diagnosis, treatment decisions, and outcomes. Differences in age of diagnosis and disease aggressiveness may be driven by genetic, socioeconomic, environmental, and systemic factors. The presence of different genetic mutations to varying rates among AA patients highlights an opportunity to prospectively evaluate these as both biomarkers of aggressive disease and therapy targets. Genetic mutation variation among patients of different ethnic origins highlights the potential for personalized screening panels to identify patients for whom mutation‐directed surveillance and treatment protocols may benefit. Uniform access to screening, diagnosis, and treatment, along with comprehensive outreach, education, and inclusion in clinical trials, is needed to improve outcomes for AA men at risk for or diagnosed with PCa.

## Author Contributions

**Charles Cobbs IV:** conceptualization (equal), data curation (equal), visualization (equal), writing – original draft (equal), writing – review and editing (equal). **Gregory T. Chesnut:** resources (equal), supervision (supporting), writing – review and editing (equal). **Ayesha A. Shafi:** conceptualization (equal), data curation (supporting), funding acquisition (equal), investigation (equal), project administration (lead), resources (equal), supervision (lead), visualization (equal), writing – original draft (equal), writing – review and editing (equal).

## Disclosure

The contents of this publication are the sole responsibility of the authors and do not necessarily reflect the views, opinions, or policies opinions of the Uniformed Services University of the Health Sciences (USUHS), Henry M. Jackson Foundation of the Advancement of Military Medicine Inc. (HJF), the Department of Defense (DoD) or the Departments of the Army, Navy, or Air Force. Mention of trade names, commercial products, or organizations does not imply endorsement by the U.S. Government.

## Ethics Statement

This review article is exempted by the Institutional Review Board.

## Conflicts of Interest

The authors declare no conflicts of interest.

## Supporting information


Table S1.

## Data Availability

The authors have nothing to report.

## References

[1] R. L. Siegel , A. N. Giaquinto , and A. Jemal , “Cancer Statistics, 2024,” CA: A Cancer Journal for Clinicians 74, no. 1 (2024): 12–49, 10.3322/caac.21820. 38230766

[2] D. A. Siegel , M. E. O'Neil , T. B. Richards , N. F. Dowling , and H. K. Weir , “Prostate Cancer Incidence and Survival, by Stage and Race/Ethnicity—United States, 2001–2017,” MMWR. Morbidity and Mortality Weekly Report 69, no. 41 (2020): 1473–1480, 10.15585/mmwr.mm6941a1. 33056955 PMC7561091

[3] R. L. Siegel , K. D. Miller , and A. Jemal , “Cancer Statistics, 2020,” CA: A Cancer Journal for Clinicians 70, no. 1 (2020): 7–30, 10.3322/caac.21590. 31912902

[4] R. L. Siegel , K. D. Miller , H. E. Fuchs , and A. Jemal , “Cancer Statistics, 2021,” CA: A Cancer Journal for Clinicians 71, no. 1 (2021): 7–33, 10.3322/caac.21654. 33433946

[5] R. L. Siegel , K. D. Miller , H. E. Fuchs , and A. Jemal , “Cancer Statistics, 2022,” CA: A Cancer Journal for Clinicians 72, no. 1 (2022): 7–33, 10.3322/caac.21708. 35020204

[6] R. L. Siegel , K. D. Miller , N. S. Wagle , and A. Jemal , “Cancer Statistics, 2023,” CA: A Cancer Journal for Clinicians 73, no. 1 (2023): 17–48, 10.3322/caac.21763. 36633525

[7] I. M. Chowdhury‐Paulino , C. Ericsson , R. Vince, Jr. , D. E. Spratt , D. J. George , and L. A. Mucci , “Racial Disparities in Prostate Cancer Among Black Men: Epidemiology and Outcomes,” Prostate Cancer and Prostatic Diseases 25, no. 3 (2022): 397–402, 10.1038/s41391-021-00451-z. 34475523 PMC8888766

[8] J. W. Lillard, Jr. , K. A. Moses , B. A. Mahal , and D. J. George , “Racial Disparities in Black Men With Prostate Cancer: A Literature Review,” Cancer 128, no. 21 (2022): 3787–3795, 10.1002/cncr.34433. 36066378 PMC9826514

[9] T. R. Rebbeck , “Prostate Cancer Disparities by Race and Ethnicity: From Nucleotide to Neighborhood,” Cold Spring Harbor Perspectives in Medicine 8, no. 9 (2018): 387, 10.1101/cshperspect.a030387. PMC6120694 29229666

[10] P. Porzycki and E. Ciszkowicz , “Modern Biomarkers in Prostate Cancer Diagnosis,” Central European Journal of Urology 73, no. 3 (2020): 300–306, 10.5173/ceju.2020.0067R. 33133657 PMC7587476

[11] M. K. David and S. W. Leslie , Prostate Specific Antigen (StatPearls, 2024). 32491427

[12] L. P. Bokhorst , C. H. Bangma , G. J. van Leenders , et al., “Prostate‐Specific Antigen‐Based Prostate Cancer Screening: Reduction of Prostate Cancer Mortality After Correction for Nonattendance and Contamination in the Rotterdam Section of the European Randomized Study of Screening for Prostate Cancer,” European Urology 65, no. 2 (2014): 329–336, 10.1016/j.eururo.2013.08.005. 23954085

[13] M. Franlund , M. Mansson , R. A. Godtman , et al., “Results From 22 Years of Followup in the Goteborg Randomized Population‐Based Prostate Cancer Screening Trial,” Journal of Urology 208, no. 2 (2022): 292–300, 10.1097/JU.0000000000002696. 35422134 PMC9275849

[14] D. Reynolds , “Prostate Cancer Screening in African American Men: Barriers and Methods for Improvement,” American Journal of Men's Health 2, no. 2 (2008): 172–177, 10.1177/1557988307312784. 19477781

[15] B. A. Mahal , M. Alshalalfa , K. H. Kensler , et al., “Racial Differences in Genomic Profiling of Prostate Cancer,” New England Journal of Medicine 383, no. 11 (2020): 1083–1085, 10.1056/NEJMc2000069. 32905685 PMC8971922

[16] Y. Han , K. A. Rand , D. J. Hazelett , et al., “Prostate Cancer Susceptibility in Men of African Ancestry at 8q24,” Journal of the National Cancer Institute 108, no. 7 (2016): djv431, 10.1093/jnci/djv431. 26823525 PMC4948565

[17] A. J. Vickers , G. Russo , H. Lilja , et al., “How Should Molecular Markers and Magnetic Resonance Imaging Be Used in the Early Detection of Prostate Cancer?,” European Urology Oncology 5, no. 2 (2022): 135–137, 10.1016/j.euo.2021.01.010. 33608234

[18] H. Y. Dong , L. Ding , T. R. Zhou , T. Yan , J. Li , and C. Liang , “FOXA1 in Prostate Cancer,” Asian Journal of Andrology 25, no. 3 (2023): 287–295, 10.4103/aja202259. 36018068 PMC10226509

[19] T. S. Lewis , P. S. Shapiro , and N. G. Ahn , “Signal Transduction Through MAP Kinase Cascades,” Advances in Cancer Research 74 (1998): 49–139, 10.1016/s0065-230x(08)60765-4. 9561267

[20] G. Pearson , F. Robinson , T. Beers Gibson , et al., “Mitogen‐Activated Protein (MAP) Kinase Pathways: Regulation and Physiological Functions,” Endocrine Reviews 22, no. 2 (2001): 153–183, 10.1210/edrv.22.2.0428. 11294822

[21] Z. Yao , R. Yaeger , V. S. Rodrik‐Outmezguine , et al., “Tumours With Class 3 BRAF Mutants Are Sensitive to the Inhibition of Activated RAS,” Nature 548, no. 7666 (2017): 234–238, 10.1038/nature23291. 28783719 PMC5648058

[22] J. R. Haling , J. Sudhamsu , I. Yen , et al., “Structure of the BRAF‐MEK Complex Reveals a Kinase Activity Independent Role for BRAF in MAPK Signaling,” Cancer Cell 26, no. 3 (2014): 402–413, 10.1016/j.ccr.2014.07.007. 25155755

[23] M. D. Fenor , S. Ruiz‐Llorente , J. F. Rodriguez‐Moreno , et al., “MEK Inhibitor Sensitivity in BRAF Fusion‐Driven Prostate Cancer,” Clinical & Translational Oncology 24, no. 12 (2022): 2432–2440, 10.1007/s12094-022-02916-6. 35994225

[24] C. Grisanzio and M. L. Freedman , “Chromosome 8q24‐Associated Cancers and MYC,” Genes & Cancer 1, no. 6 (2010): 555–559, 10.1177/1947601910381380. 21779458 PMC3092220

[25] Z. Szallasi , M. Diossy , V. Tisza , et al., “Increased Frequency of CHD1 Deletions in Prostate Cancers of African American Men Is Associated With Rapid Disease Progression Without Inducing Homologous Recombination Deficiency,” Research Square, 2024, 10.21203/rs.3.rs-3,995,251/v1.

[26] V. Kari , W. Y. Mansour , S. K. Raul , et al., “Loss of CHD1 Causes DNA Repair Defects and Enhances Prostate Cancer Therapeutic Responsiveness,” EMBO Reports 17, no. 11 (2016): 1609–1623, 10.15252/embr.201642352. 27596623 PMC5090703

[27] T. R. Shenoy , G. Boysen , M. Y. Wang , et al., “CHD1 Loss Sensitizes Prostate Cancer to DNA Damaging Therapy by Promoting Error‐Prone Double‐Strand Break Repair,” Annals of Oncology 28, no. 7 (2017): 1495–1507, 10.1093/annonc/mdx165. 28383660 PMC5834074

[28] H. Li , L. Gigi , and D. Zhao , “CHD1, a Multifaceted Epigenetic Remodeler in Prostate Cancer,” Frontiers in Oncology 13 (2023): 1123362, 10.3389/fonc.2023.1123362. 36776288 PMC9909554

[29] J. Gong , D. M. Kim , M. R. Freeman , et al., “Genetic and Biological Drivers of Prostate Cancer Disparities in Black Men,” Nature Reviews Urology 21 (2023): 274–289, 10.1038/s41585-023-00828-w. 37964070

[30] N. Zaman , P. N. Giannopoulos , S. Chowdhury , et al., “Proteomic‐Coupled‐Network Analysis of T877A‐Androgen Receptor Interactomes Can Predict Clinical Prostate Cancer Outcomes Between White (Non‐Hispanic) and African‐American Groups,” PLoS One 9, no. 11 (2014): e113190, 10.1371/journal.pone.0113190. 25409505 PMC4237393

[31] R. A. Karunamuni , M. P. Huynh‐Le , C. C. Fan , et al., “African‐Specific Improvement of a Polygenic Hazard Score for Age at Diagnosis of Prostate Cancer,” International Journal of Cancer 148, no. 1 (2021): 99–105, 10.1002/ijc.33282. 32930425 PMC8135907

[32] F. Chen , R. K. Madduri , A. A. Rodriguez , et al., “Evidence of Novel Susceptibility Variants for Prostate Cancer and a Multiancestry Polygenic Risk Score Associated With Aggressive Disease in Men of African Ancestry,” European Urology 84, no. 1 (2023): 13–21, 10.1016/j.eururo.2023.01.022. 36872133 PMC10424812

[33] S. Awasthi , A. Berglund , J. Abraham‐Miranda , et al., “Comparative Genomics Reveals Distinct Immune‐Oncologic Pathways in African American Men With Prostate Cancer,” Clinical Cancer Research 27, no. 1 (2021): 320–329, 10.1158/1078-0432.CCR-20-2925. 33037017 PMC8042600

[34] M. Gillard , R. Javier , Y. Ji , et al., “Elevation of Stromal‐Derived Mediators of Inflammation Promote Prostate Cancer Progression in African‐American Men,” Cancer Research 78, no. 21 (2018): 6134–6145, 10.1158/0008-5472.CAN-17-3810. 30181178

[35] M. A. Kinseth , Z. Jia , F. Rahmatpanah , et al., “Expression Differences Between African American and Caucasian Prostate Cancer Tissue Reveals That Stroma Is the Site of Aggressive Changes,” International Journal of Cancer 134, no. 1 (2014): 81–91, 10.1002/ijc.28326. 23754304 PMC3800217

[36] G. C. Gee and C. L. Ford , “Structural Racism and Health Inequities: Old Issues, New Directions,” Du Bois Review: Social Science Research on Race 8, no. 1 (2011): 115–132, 10.1017/S1742058X11000130. 25632292 PMC4306458

[37] T. E. Byers , H. J. Wolf , K. R. Bauer , et al., “The Impact of Socioeconomic Status on Survival After Cancer in the United States: Findings From the National Program of Cancer Registries Patterns of Care Study,” Cancer 113, no. 3 (2008): 582–591, 10.1002/cncr.23567. 18613122

[38] K. Schwartz , I. J. Powell , W. Underwood, 3rd , J. George , C. Yee , and M. Banerjee , “Interplay of Race, Socioeconomic Status, and Treatment on Survival of Patients With Prostate Cancer,” Urology 74, no. 6 (2009): 1296–1302, 10.1016/j.urology.2009.02.058. 19962532 PMC2791874

[39] H. T. Vigneswaran , J. S. Jagai , D. T. Greenwald , et al., “Association Between Environmental Quality and Prostate Cancer Stage at Diagnosis,” Prostate Cancer and Prostatic Diseases 24, no. 4 (2021): 1129–1136, 10.1038/s41391-021-00370-z. 33947975

[40] H. S. Iyer , L. Valeri , P. James , et al., “The Contribution of Residential Greenness to Mortality Among Men With Prostate Cancer: A Registry‐Based Cohort Study of Black and White Men,” Environmental Epidemiology 4, no. 2 (2020): e087, 10.1097/EE9.0000000000000087. 32337472 PMC7147390

[41] Y. Wu , M. Sarkissyan , and J. V. Vadgama , “Epigenetics in Breast and Prostate Cancer,” Methods in Molecular Biology 1238 (2015): 425–466, 10.1007/978-1-4939-1804-1_23. 25421674 PMC4364390

[42] K. Woodson , R. Hayes , L. Wideroff , L. Villaruz , and J. Tangrea , “Hypermethylation of GSTP1, CD44, and E‐Cadherin Genes in Prostate Cancer Among US Blacks and Whites,” Prostate 55, no. 3 (2003): 199–205, 10.1002/pros.10236. 12692786

[43] J. Cullen , S. A. Brassell , Y. Chen , et al., “Racial/Ethnic Patterns in Prostate Cancer Outcomes in an Active Surveillance Cohort,” Prostate Cancer 2011 (2011): 234519, 10.1155/2011/234519. 22096650 PMC3195388

[44] G. Chornokur , K. Dalton , M. E. Borysova , and N. B. Kumar , “Disparities at Presentation, Diagnosis, Treatment, and Survival in African American Men, Affected by Prostate Cancer,” Prostate 71, no. 9 (2011): 985–997, 10.1002/pros.21314. 21541975 PMC3083484

[45] A. J. Vickers , B. Mahal , and O. O. Ogunwobi , “Racism Does Not Cause Prostate Cancer, It Causes Prostate Cancer Death,” Journal of Clinical Oncology 41, no. 12 (2023): 2151–2154, 10.1200/JCO.22.02203. 36693227 PMC10448930

[46] D. J. George , S. Halabi , E. I. Heath , et al., “A Prospective Trial of Abiraterone Acetate Plus Prednisone in Black and White Men With Metastatic Castrate‐Resistant Prostate Cancer,” Cancer 127, no. 16 (2021): 2954–2965, 10.1002/cncr.33589. 33951180 PMC9527760

[47] J. Gong , D. M. Kim , A. M. De Hoedt , et al., “Disparities With Systemic Therapies for Black Men Having Advanced Prostate Cancer: Where Do We Stand?,” Journal of Clinical Oncology 42, no. 2 (2024): 228–236, 10.1200/JCO.23.00949. 37890125 PMC10824384

[48] S. Awasthi , G. D. Grass , J. Torres‐Roca , et al., “Genomic Testing in Localized Prostate Cancer Can Identify Subsets of African Americans With Aggressive Disease,” Journal of the National Cancer Institute 114, no. 12 (2022): 1656–1664, 10.1093/jnci/djac162. 36053178 PMC9745424

[49] C. K. Tsao , J. Sfakianos , B. Liaw , et al., “Phase II Trial of Abiraterone Acetate Plus Prednisone in Black Men With Metastatic Prostate Cancer,” Oncologist 21, no. 12 (2016): 1414.e9, 10.1634/theoncologist.2016-0026. 27742908 PMC5153336

[50] A. P. Cole , P. Herzog , H. S. Iyer , et al., “Racial Differences in the Treatment and Outcomes for Prostate Cancer in Massachusetts,” Cancer 127, no. 15 (2021): 2714–2723, 10.1002/cncr.33564. 33999405 PMC9107927

[51] D. A. Barocas , R. Grubb, 3rd , A. Black , et al., “Association Between Race and Follow‐Up Diagnostic Care After a Positive Prostate Cancer Screening Test in the Prostate, Lung, Colorectal, and Ovarian Cancer Screening Trial,” Cancer 119, no. 12 (2013): 2223–2229, 10.1002/cncr.28042. 23559420

[52] J. Lin , D. Nousome , J. Jiang , G. T. Chesnut , C. D. Shriver , and K. Zhu , “Five‐Year Survival of Patients With Late‐Stage Prostate Cancer: Comparison of the Military Health System and the U.S. General Population,” British Journal of Cancer 128, no. 6 (2023): 1070–1076, 10.1038/s41416-022-02136-3. 36609596 PMC10006403

[53] N. Oehrlein , S. A. Streicher , H. C. Kuo , et al., “Race‐Specific Prostate Cancer Outcomes in a Cohort of Military Health Care Beneficiaries Undergoing Surgery: 1990–2017,” Cancer Medicine 11, no. 22 (2022): 4354–4365, 10.1002/cam4.4787. 35638719 PMC9678085

[54] J. Pincus , J. W. Greenberg , C. Natale , et al., “Five‐Year Prospective Observational Study of African‐American Men on Active Surveillance for Prostate Cancer Demonstrates Race Is Not Predictive of Oncologic Outcomes,” Oncologist 28, no. 2 (2023): 149–156, 10.1093/oncolo/oyac154. 35920550 PMC9907040

[55] R. Deka , P. T. Courtney , J. K. Parsons , et al., “Association Between African American Race and Clinical Outcomes in Men Treated for Low‐Risk Prostate Cancer With Active Surveillance,” JAMA 324, no. 17 (2020): 1747–1754, 10.1001/jama.2020.17020. 33141207 PMC7610194

[56] K. H. Kensler , C. H. Pernar , B. A. Mahal , et al., “Racial and Ethnic Variation in PSA Testing and Prostate Cancer Incidence Following the 2012 USPSTF Recommendation,” Journal of the National Cancer Institute 113, no. 6 (2021): 719–726, 10.1093/jnci/djaa171. 33146392 PMC8168268

[57] M. A. Hudson , S. Luo , T. Chrusciel , et al., “Do Racial Disparities Exist in the Use of Prostate Cancer Screening and Detection Tools in Veterans?,” Urologic Oncology 32, no. 1 (2014): 34.e9–34.18, 10.1016/j.urolonc.2013.01.003. PMC4417353 23506962

[58] S. A. Brassell , A. Dobi , G. Petrovics , S. Srivastava , and D. McLeod , “The Center for Prostate Disease Research (CPDR): A Multidisciplinary Approach to Translational Research,” Urologic Oncology 27, no. 5 (2009): 562–569, 10.1016/j.urolonc.2009.01.023. 19720304

[59] C. H. Halbert , B. Weathers , E. Delmoor , et al., “Racial Differences in Medical Mistrust Among Men Diagnosed With Prostate Cancer,” Cancer 115, no. 11 (2009): 2553–2561, 10.1002/cncr.24249. 19296516 PMC2701108

[60] C. R. Rogers , M. J. Rovito , M. Hussein , et al., “Attitudes Toward Genomic Testing and Prostate Cancer Research Among Black Men,” American Journal of Preventive Medicine 55, no. 5 Suppl 1 (2018): S103–S111, 10.1016/j.amepre.2018.05.028. 30670195 PMC6352989

[61] B. L. Kinlock , R. J. Thorpe, Jr. , D. L. Howard , et al., “Racial Disparity in Time Between First Diagnosis and Initial Treatment of Prostate Cancer,” Cancer Control 23, no. 1 (2016): 47–51, 10.1177/107327481602300108. 27009456 PMC6448564

[62] O. T. Hall , N. M. Bhadra‐Heintz , J. Teater , et al., “Group‐Based Medical Mistrust and Care Expectations Among Black Patients Seeking Addiction Treatment,” Drug and Alcohol Dependence Reports 2 (2022): 100026, 10.1016/j.dadr.2022.100026. 36845897 PMC9949334

[63] D. H. Thom , K. M. Ribisl , A. L. Stewart , and D. A. Luke , “Further Validation and Reliability Testing of the Trust in Physician Scale. The Stanford Trust Study Physicians,” Medical Care 37, no. 5 (1999): 510–517, 10.1097/00005650-199905000-00010. 10335753

[64] T. Hewitt , K. A. Killinger , S. Hiller , J. A. Boura , and M. Lutz , “Exploring Racial Differences Surrounding Prostate Cancer Screening: Beliefs and Attitudes in Community Dwelling Men Attending an Urban Men's Health Event,” American Journal of Men's Health 12, no. 6 (2018): 1929–1936, 10.1177/1557988318784838. PMC6199456 29952245

[65] B. E. Gordon , R. Basak , W. R. Carpenter , D. Usinger , P. A. Godley , and R. C. Chen , “Factors Influencing Prostate Cancer Treatment Decisions for African American and White Men,” Cancer 125, no. 10 (2019): 1693–1700, 10.1002/cncr.31932. 30695113 PMC6604809

[66] L. M. Hurwitz , J. Cullen , S. Elsamanoudi , et al., “A Prospective Cohort Study of Treatment Decision‐Making for Prostate Cancer Following Participation in a Multidisciplinary Clinic,” Urologic Oncology 34, no. 5 (2016): 233.e17–233.e25, 10.1016/j.urolonc.2015.11.014. 26705101

[67] B. F. Drake , T. E. Keane , C. M. Mosley , et al., “Prostate Cancer Disparities in South Carolina: Early Detection, Special Programs, and Descriptive Epidemiology,” Journal of the South Carolina Medical Association 102, no. 7 (2006): 241–249, https://www.ncbi.nlm.nih.gov/pubmed/17319238. 17319238

[68] A. I. Vines , J. C. Hunter , V. A. Carlisle , and A. N. Richmond , “Prostate Cancer Ambassadors: Process and Outcomes of a Prostate Cancer Informed Decision‐Making Training Program,” American Journal of Men's Health 11, no. 1 (2017): 54–62, 10.1177/1557988316644979. PMC5675173 27099348

[69] S. H. Meghani , J. M. Brooks , T. Gipson‐Jones , R. Waite , L. Whitfield‐Harris , and J. A. Deatrick , “Patient‐Provider Race‐Concordance: Does It Matter in Improving Minority Patients' Health Outcomes?,” Ethnicity & Health 14, no. 1 (2009): 107–130, 10.1080/13557850802227031. 19012091 PMC3209820

[70] B. C. Tilley , A. G. Mainous, 3rd , R. P. Amorrortu , et al., “Using Increased Trust in Medical Researchers to Increase Minority Recruitment: The RECRUIT Cluster Randomized Clinical Trial,” Contemporary Clinical Trials 109 (2021): 106519, 10.1016/j.cct.2021.106519. 34333138 PMC8665835

[71] J. L. Beebe‐Dimmer , J. J. Ruterbusch , K. A. Cooney , et al., “Racial Differences in Patterns of Treatment Among Men Diagnosed With De Novo Advanced Prostate Cancer: A SEER‐Medicare Investigation,” Cancer Medicine 8, no. 6 (2019): 3325–3335, 10.1002/cam4.2092. 31094098 PMC6558501

[72] P. Riviere , E. Luterstein , A. Kumar , et al., “Survival of African American and Non‐Hispanic White Men With Prostate Cancer in an Equal‐Access Health Care System,” Cancer 126, no. 8 (2020): 1683–1690, 10.1002/cncr.32666. 31984482

[73] T. Rude , D. Walter , S. Ciprut , et al., “Interaction Between Race and Prostate Cancer Treatment Benefit in the Veterans Health Administration,” Cancer 127, no. 21 (2021): 3985–3990, 10.1002/cncr.33643. 34184271

[74] S. P. Weinrich , “Prostate Cancer Screening in High‐Risk Men: African American Hereditary Prostate Cancer Study Network,” Cancer 106, no. 4 (2006): 796–803, 10.1002/cncr.21674. 16411222

[75] G. Aurilio , A. Cimadamore , R. Mazzucchelli , et al., “Androgen Receptor Signaling Pathway in Prostate Cancer: From Genetics to Clinical Applications,” Cells 9, no. 12 (2020): 2653, 10.3390/cells9122653. 33321757 PMC7763510

