# Supplementary material for: Understanding Racial Disparities in Prostate Cancer: A Multifaceted Approach
Source: Cancer Med. 2025 May 30;14(11):e70979. doi: 10.1002/cam4.70979 (PMC12123386; doi:10.1002/cam4.70979)
Supplement: Supplementary file 1 — Table S1. [file CAM4-14-e70979-s001.docx]

**Supplementary Table 1. Outcomes when AA men are provided similar care to CA males**

| Study | Summary |
| --- | --- |
| **Five-year survival of patients with late-stage PCA: comparison of the Military Health System and the U.S. general population** | Men with advanced PCa tumors revealed that patients in the military health system had improved survival compared to their counterparts in the U.S. general population, and the survival advantage was consistently observed in all patients, regardless of race. |
| **Race‐specific PCa outcomes in a cohort of military health care beneficiaries undergoing surgery: 1990–2017** | Despite AA men having shorter survival time from radical prostatectomy to biochemical recurrence (BCR), the group showed comparable survival time from BCR to metastasis and metastasis to overall death |
| **Differences in rates of pelvic lymph node dissection in National Comprehensive Cancer Network (NCCN) favorable, unfavorable intermediate- and high-risk PCa across United States SEER registries** | Examining patients with PCa in those with a high enough severity to warrant lymph node dissection, the rates of those who did not have this measure taken was similar for AA and CA men. |
| **Five-Year Prospective Observational Study of AA Men on Active Surveillance for PCa Demonstrates Race Is Not Predictive of Oncologic Outcomes** | Race was not predictive of Gleason grade progression, active surveillance discontinuation, or biochemical recurrence in a cohort with a majority of AA men^1^ |
| **Association Between AA Race and Clinical Outcomes in Men Treated for Low-Risk PCa With Active Surveillance** | AA men had a higher incidence of disease progression and definitive treatment compared to CA men, but no significant differences in metastasis or PCa-specific mortality |

**References:**

1. Pincus, J., Greenberg, J. W., Natale, C. et al.: Five-Year Prospective Observational Study of African-American Men on Active Surveillance for Prostate Cancer Demonstrates Race Is Not Predictive of Oncologic Outcomes. Oncologist, **28:** 149, 2023
